# Sex difference analyses under scrutiny

**DOI:** 10.7554/eLife.74135

**Published:** 2021-11-02

**Authors:** Colby J Vorland

**Affiliations:** 1 Department of Applied Health Science, Indiana University School of Public Health Bloomington United States

**Keywords:** meta-research, sex inclusion, sex differences, statistics, study design, methodological weakness, None

## Abstract

A survey reveals that many researchers do not use appropriate statistical analyses to evaluate sex differences in biomedical research.

**Related research article** Garcia-Sifuentes Y, Maney DL. 2021. Reporting and misreporting of sex differences in the biological sciences. *eLife* **10**:e70817. doi: 10.7554/eLife.70817

Scientific research requires the use of appropriate methods and statistical analyses, otherwise results and interpretations can be flawed. How research outcomes differ by sex, for example, has historically been understudied, and only recently have policies been implemented to require such consideration in the design of a study (e.g., NIH, 2015).

Over two decades ago, the renowned biomedical statistician Doug Altman labeled methodological weaknesses a “scandal”, raising awareness of shortcomings related to the representativeness of research as well as inappropriate research designs and statistical analysis (Altman, 1994). These methodological weaknesses extend to research on sex differences: simply adding female cells, animals, or participants to experiments does not guarantee an improved understanding of this field of research. Rather, the experiments must also be correctly designed and analyzed appropriately to examine such differences. While guidance exists for proper analysis of sex differences, the frequency of errors in published research articles related to this topic has not been well understood (e.g., Beltz et al., 2019).

Now, in eLife, Yesenia Garcia-Sifuentes and Donna Maney of Emory University fill this gap by surveying the literature to examine whether the statistical analyses used in different research articles are appropriate to support conclusions of sex differences (Garcia-Sifuentes and Maney, 2021). Drawing from a previous study that surveyed articles studying mammals from nine biological disciplines, Garcia-Sifuentes and Maney sampled 147 articles that included both males and females and performed an analysis by sex (Woitowich et al., 2020).

Over half of the articles surveyed (83, or 56%) reported a sex difference. Garcia-Sifuentes and Maney examined the statistical methods used to analyze sex differences and found that over a quarter (24 out of 83) of these articles did not perform or report a statistical analysis supporting the claim of a sex difference. A factorial design with sex as a factor is an appropriate way to examine sex differences in response to treatment, by giving each sex each treatment option (such as a treatment or control diet; see Figure 1A). A slight majority of all articles (92, or 63%) used a factorial design. Within the articles using a factorial design, however, less than one third (27) applied and reported a method appropriate to test for sex differences (e.g., testing for an interaction between sex and the exposure, such as different diets; Figure 1B). Similarly, within articles that used a factorial design and concluded a sex-specific effect, less than one third (16 out of 53) used an appropriate analysis.

**Figure 1.**
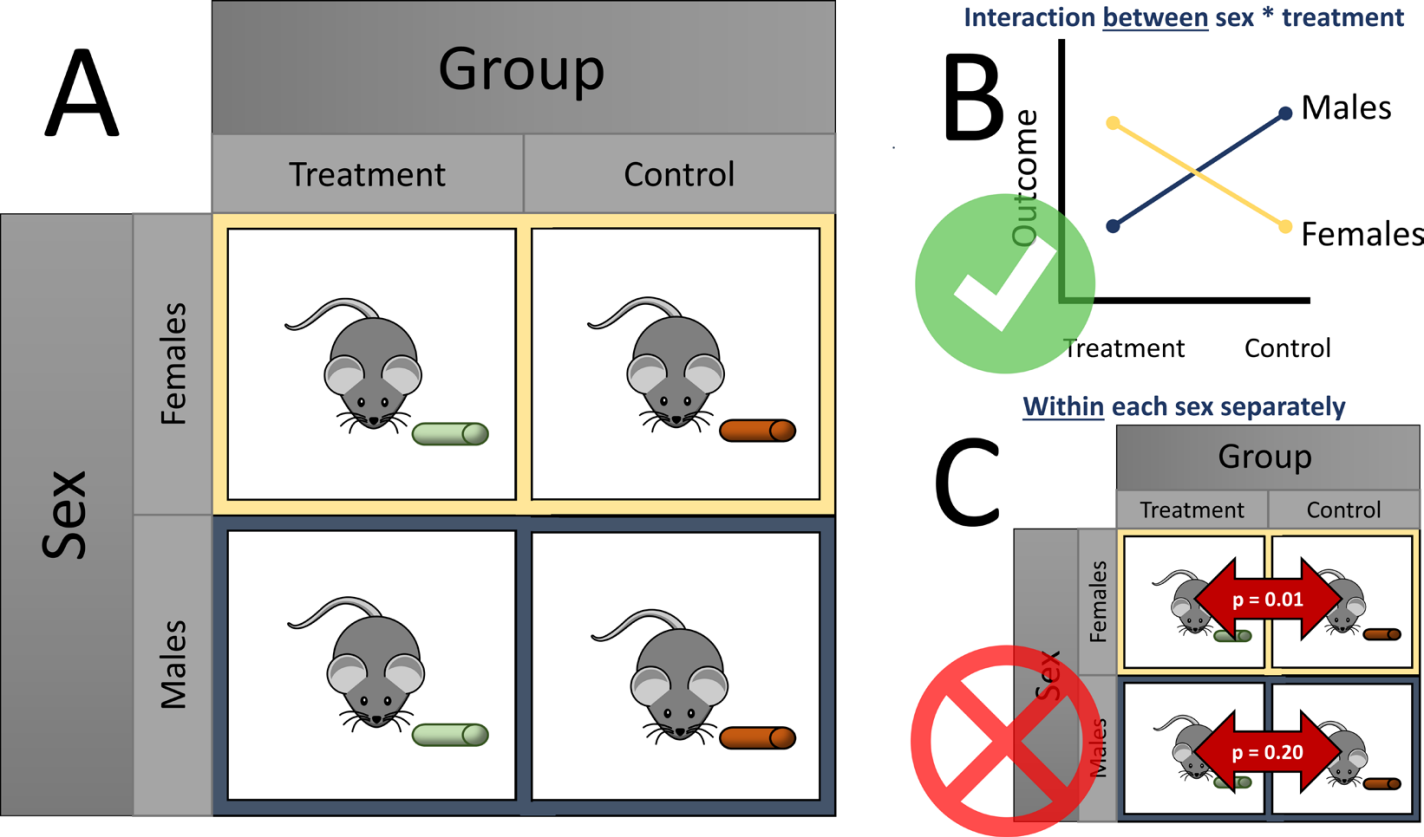
Considering sex differences in experimental design. (**A**) A so-called factorial design permits testing of sex differences. For example, both female (yellow boxes) and male mice (blue boxes) are fed either a treatment diet (green pellets) or control diet (orange pellets). Garcia-Sifuentes and Maney found that 63 % of articles employed a factorial design in at least one experiment with sex as a factor. (**B**) An appropriate way to statistically test for sex differences is with a two-way analysis of variance (ANOVA). If a statistically significant interaction is observed between sex and treatment, as shown in the figure, evidence for a sex difference is supported. Garcia-Sifuentes and Maney found that in studies using a factorial design, less than one third tested for an interaction between sex and treatment. (**C**) Performing a statistical test between the treatment and control groups within each sex, and comparing the nominal statistical significance, is not a valid method to look for sex differences. Yet, this method was used in nearly half of articles that used a factorial design and concluded a sex-specific effect.

Notably, nearly half of the articles (24 out of 53) that concluded a sex-specific effect statistically tested the effect of treatment within each sex and compared the resulting statistical significance. In other words, when one sex had a statistically significant change and the other did not, the authors of the original studies concluded that a sex difference existed. This approach, which is sometimes called ‘differences in nominal significance’, or ‘DINS’ error (George et al., 2016), is invalid and has been found to occur for decades among several disciplines, including neuroscience (Nieuwenhuis et al., 2011), obesity and nutrition (Bland and Altman, 2015; George et al., 2016; Vorland et al., 2021), and more general areas (Gelman and Stern, 2006; Makin, 2019; Matthews and Altman, 1996; Sainani, 2010; Figure 1C).

This approach is invalid because testing within each sex separately inflates the probability of falsely concluding that a sex-specific effect is present compared to testing between them directly. Other inappropriate analyses that were identified in the survey included testing sex within treatment and ignoring control animals; not reporting results after claiming to do an appropriate analysis; or claiming an effect when the appropriate analysis was not statistically significant despite subscribing to ‘null hypothesis significance’ testing. Finally, when articles pooled the data of males and females together in their analysis, about half of them did not first test for a sex difference, potentially masking important differences.

The results of Garcia-Sifuentes and Maney highlight the need for thoughtful planning of study design, analysis, and communication to maximize our understanding and use of biological sex differences in practice. Although the survey does not quantify what proportion of this research comes to incorrect conclusions from using inappropriate statistical methods, which would require estimation procedures or reanalyzing the data, many of these studies’ conclusions may change if they were analyzed correctly. Misleading results divert our attention and resources, contributing to the larger problem of ‘waste’ in biomedical research, that is, the avoidable costs of research that does not contribute to our understanding of what is true because it is flawed, methodologically weak, or not clearly communicated (Glasziou and Chalmers, 2018).

What can the scientific enterprise do about this problem? The survey suggests that there may be a large variability in discipline-specific practices in the design, reporting, and analysis strategies to examine sex differences. Although larger surveys are needed to assess these more comprehensively, they may imply that education and support efforts could be targeted where they are most needed. Compelling scientists to publicly share their data can facilitate reanalysis when statistical errors are discovered – though the burden on researchers performing the reanalysis is not trivial. Partnering with statisticians in the design, analysis, and interpretation of research is perhaps the most effective means of prevention.

Scientific research often does not reflect the diversity of those who benefit from it. Even when it does, using methods that are inappropriate fails to support the progress toward equity. Surely this is nothing less than a scandal.
